# Carcinoma

**Published:** 1906-08-11

**Authors:** 


### CARCINOMA.

(*Continued from page* 124.)

**On the Treatment of Tuberculosis of the Bones and Joints.**—In the discussion [4] on the treatment of tubercular joints at the recent International Surgical Congress there was a surprising advocacy of conservative measures. All are agreed on two principles—namely, those of rest and of hygienic treatment as having the greatest tendency to promote recovery. Tubercular disease is one in which natural healing can and will take place if only the general and local conditions are favourable. Kocher was the only speaker who expressed himself in favour of early operation in all cases. But in addition to immobilisation of the joint and good surroundings for the patient there are three local measures strongly advocated by various authorities as hastening the process of cure. Bier obtains the best results from his method of passive congestion induced by means of a rubber band applied on the proximal side of the joint. The band is applied for one to three hours a day, and should not be tight enough to cause pain. The method is employed even in the presence of sinuses, the dressings of which are removed whilst the treatment is being carried out. If an abscess occurs during treatment it should be aspirated or opened by a small incision. All ages are suitable for this treatment, and the only contra-indications to it in the opinion of its originator are general conditions which demand amputation or the ankylosis of a joint in a vicious attitude which requires excision. Except in the cases of the knee and ankle where ankylosis is to be expected, passive movements and gentle massage should be combined with the treatment in order to ensure a movable joint. In the case of the wrist Bier reports 17 cases in which 15 were cured with a movable joint, the mean period of treatment being about one year. In the elbow 8 cases were cured and 3 improved, in a total of 11, the time taken being nine months. In the ankle 8 were cured and 3 improved, out of a total of 13, the treatment occupying ten months. In the knee, however, this method is unsatisfactory, and its author could only bring forward 3 cases in which it had proved beneficial, whereas in 8 cases in which he had tried it he had to resort to resection. And, in general, other surgeons who have tried this method of passive congestion are not so favourably impressed with it. Then there is the method of antiseptic injection which is favoured by many surgeons. Broca advises the use of one-in-ten solution of iodoform in ether, which is injected into the joint after the aspiration of the fluid contained in it, and this method is especially valuable in cases of tubercular hydrarthrosis in children. And in the third place Lannelongne's counter-irritation by deep interstitial injections of chloride of zinc is highly spoken of. It is supposed to act by producing fibrosis of the structures surrounding the tubercular focus, and so hastening the natural process of cure. Cumston [5] speaks highly of it in treating the extra articular tubercular lesions found in the epiphises. A solution of 5 to 10 per cent. is injected by a hypodermic needle, not into the tubercular focus, but into the tissues surrounding it, a few drops of the solution being introduced into each point. But the method is one which involves much pain and a certain amount of risk of damage to the soft tissues. In the cases of the hip and the knee opinion seems to be very uniform. In the former, unless suppuration has taken place, rest and immobilisation are nearly always efficient, and radical operations will only be required when a sequestrum has to be removed or an abscess opened. In the knee all favour operation in the majority of cases, although conservative measures may be tried for a reasonable time in patients under fifteen years of age. In adults resection should be resorted to without delay, and gives very good results.

**On the Philo-phlogistic Treatment of Inflammation.**—Biers'[6] method of passive hyperæmia mentioned above is also applied to other than tubercular lesions and in situations elsewhere than in the limbs. The principle on which the method is based is the encouragement of leucocytosis by inducing and encouraging to the utmost extent the local condition of hyperæmia around the lesion. The appearance of the part after the method has been in application for some time is very remarkable, being swollen, fiery red, and even œdematous. The patient, however, is quite comfortable, and the healing process is greatly accelerated. If pus forms, a small stab incision is all that is necessary to evacuate it, however large the amount. For the breast and the surface of the trunk, the hyperæmia is induced by means of large-sized cupping glasses, the contained air being exhausted to the extent required by means of an aspirating pump. The method has even been applied to the head itself by placing the constricting bandage round the throat. It is said rapidly to cut short an attack of coryza, and even cases of middle-ear disease are cured by it.

[4] Edin. Med. Jour., Jan. 1906. [5] Dublin Jour. of Med. Sci., Nov. 1, 1905. [6] Practitioner, Sept. 1905.

